# Diet and Prey Preference of Tigers (*Panthera tigris*) in and Around Chitwan National Park, Nepal

**DOI:** 10.1002/ece3.73409

**Published:** 2026-04-08

**Authors:** Hari Bhadra Acharya, Laura D. Bertola, Dinesh Neupane, Babu Ram Lamichhane, Herwig Leirs, Prajwol Manandhar, Hans H. de Iongh

**Affiliations:** ^1^ Ministry of Forests and Environment Kathmandu Nepal; ^2^ University of Antwerp Antwerp Belgium; ^3^ National Centre for Biological Sciences Tata Institute of Fundamental Research Bangalore India; ^4^ Zoological Society of London Kathmandu Nepal; ^5^ Wildlife Conservation and Research Endeavour (WILD CARE) Lalitpur Nepal; ^6^ Center for Molecular Dynamics Nepal Kathmandu Nepal

**Keywords:** DNA metabarcoding, human‐wildlife conflict, Jacob's index, microscopic hair analysis, relative biomass, scats

## Abstract

Assessing the diet of apex predators such as tiger is important, as it provides key ecological information on prey availability and preferences. It helps to understand the effects of conservation strategy and secure the long‐term survival of tigers in the area. In this research, we identified the diet composition of tigers via DNA metabarcoding and microscopy analysis of scat samples collected during the dry season of 2024 in Nepal's Chitwan National Park and Buffer Zone area. The scat analysis showed spotted deer dominated the tiger diet, comprising the highest frequency and percentage of occurrences in scats with greater relative biomass consumed. No trace of livestock was detected in the scats analyzed. Specifically, tigers preferred medium‐sized prey (e.g., hog deer, wild boar, and spotted deer). The study identified seven prey species in the tiger diet analyzed through DNA metabarcoding and microscopic hair analysis; however, the type and proportion of their contents varied between the methods. The diet composition of tigers in the national park comprised a significantly higher number of species (*n* = 7) than in the buffer zone area (*n* = 3), indicating the park as a better tiger habitat providing a variety of prey (diet) available in the park with a higher opportunity of food choices. This study provides important insights for the population recovery of tigers in protected areas, improving prey abundance in the natural habitat, and limiting human‐tiger conflict in the buffer zone.

## Graphical abstract

Spotted deer dominated the tiger diet in Chitwan National Park and Buffer Zone area, Nepal aligned with the highest prey density in this area. However, tigers preferred medium‐sized prey (e.g., hog deer, wild boar, and spotted deer). The diet composition of tigers in the national park comprised a significantly higher number of species (*n* = 7) than in the buffer zone area (*n* = 3), indicating the park as a better tiger habitat providing a variety of prey (diet) available in the park with higher opportunity of food choice.

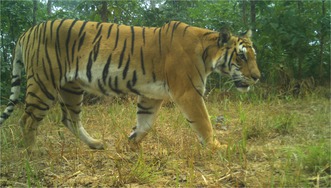

## 1 Introduction

The tiger ( *Panthera tigris* ) is the largest feline in the world, occupying a diversity of habitats, ranging from semi‐arid areas in India to the tropical rain forests of Sumatra (Indonesia) to the coniferous forests of Russia (Seidensticker et al. 1999; Sunquist 1999). Tigers are pivotal to ecosystem functions, providing key ecological services such as controlling food webs, facilitating nutrient cycling, and impeding exotic species invasions (Hammerschlag et al. 2019). Tigers are territorial, solitary hunters, well‐equipped to ambush and capture prey (Karanth and Sunquist 1995). However, loss of habitat, poaching, and depletion of prey are major threats to tigers (Goodrich et al. 2015).

Thorough understanding of prey consumption by tigers helps us in evaluating its role in an ecosystem, which is crucial in developing appropriate management strategies for their conservation and mitigating human–tiger conflict (Chakrabarti et al. 2016). However, the elusive nature and low population densities of large cats like tigers are a constraint to observing predation and their feeding activities in the natural habitat (Wang et al. 2022). Thus, the non‐invasive method of sample collection through feces can facilitate not only ecological, genetic, and physiological studies but also the diet composition of these elusive species (Ramakrishnan et al. 1999; Sarkar et al. 2018).

The diets and food habits of tigers across different areas have site‐specific reports ranging from small to large‐sized wild prey and livestock to their diets (Karanth and Sunquist 1995; Støen and Wegge 1996; Bhattarai and Kindlmann 2012; Ramesh et al. 2012; Bhandari et al. 2017, Bhandari et al. 2025; Reynaert 2018; Upadhyaya et al. 2018). Many studies suggested mainly of medium‐sized prey species (50–100 kg), such as spotted deer ( *Axis axis* ), wild boar ( *Sus scrofa* ), barking deer ( *Muntiacus muntjak* ), and large‐sized prey species (> 100 kg), such as sambar ( *Rusa unicolor* ), gaur ( *Bos gaurus* ), and others in the tiger diets (Karanth and Sunquist 1995; Bhattarai and Kindlmann 2012). Some other studies suggested that subadult tigers and male tigers killed more livestock than wild prey, even when wild prey was available (Kolipaka 2018). Understanding tiger diets and prey preferences provides an insight into identifying the habitat management priority for the maintenance of the tiger population inside the national park (Kolipaka 2018).

The population of tigers in Nepal has increased in and around the tiger‐bearing national parks during the past decade (DNPWC and DFSC 2022). With an increasing population, few tigers are dispersed to the forests adjoining the national parks, where the forests are proximate to human settlements (Acharya et al. 2026 in press). However, communities' natural resource dependency and forest‐dwelling activities for fodder and fuel collection have resulted in an increase in the human‐tiger interface, even if tigers are inside a national park or forest (Bhattarai et al. 2019). This trend of rising tiger population may push more tigers into peripheral habitats with chances of higher interaction between tigers and local communities that may cause more incidents of livestock predation and attacks on humans (Kapfer et al. 2011). Tigers occurring in human‐dominated landscapes are typically either injured or older individuals that have been displaced by dominant animals, or transient dispersing individuals that have not yet established a territory (Lamichhane et al. 2017).

As the Chitwan tiger population increases, more subadults and transient tigers are likely to disperse out of core areas and come into conflict with local people (Lamichhane et al. 2017). A comprehensive study is required to understand the dietary composition, including prey preference of tigers, in the context of population recovery, with the possibility of human‐tiger conflicts in and around the national park.

However, traditional microscopic hair analysis techniques are widely used to identify the prey species; molecular tools are also suitable to identify the species through non‐invasive techniques of scat analysis and have already provided important insights into the tiger population structure in Nepal (Thapa et al. 2018). Specifically for diet studies, DNA metabarcoding has been shown to provide an accurate picture of diet composition (Shehzad et al. 2012; Thuo et al. 2019; Groen et al. 2023).

We aimed to assess the dietary composition and prey preference of tigers in Chitwan National Park and Buffer Zone area, Nepal, using both microscopy and molecular techniques. The specific research objectives were to: (1) identify the contribution of prey species to the tiger diet and its prey preferences; (2) compare the differences in diet composition of tigers between the national park and buffer zone area; and (3) evaluate the performance of DNA metabarcoding and microscopic hair analysis methods for assessing the tiger diets.

The results of this study will help in understanding the in‐depth of tiger diets and prey preferences that can inform the likelihood of conflicts and contribute to proper management of tigers in a human‐dominated landscape by understanding the tiger diet and prey preference.

## 2 Materials and Methods

### 2.1 Study Area

**FIGURE 1:**
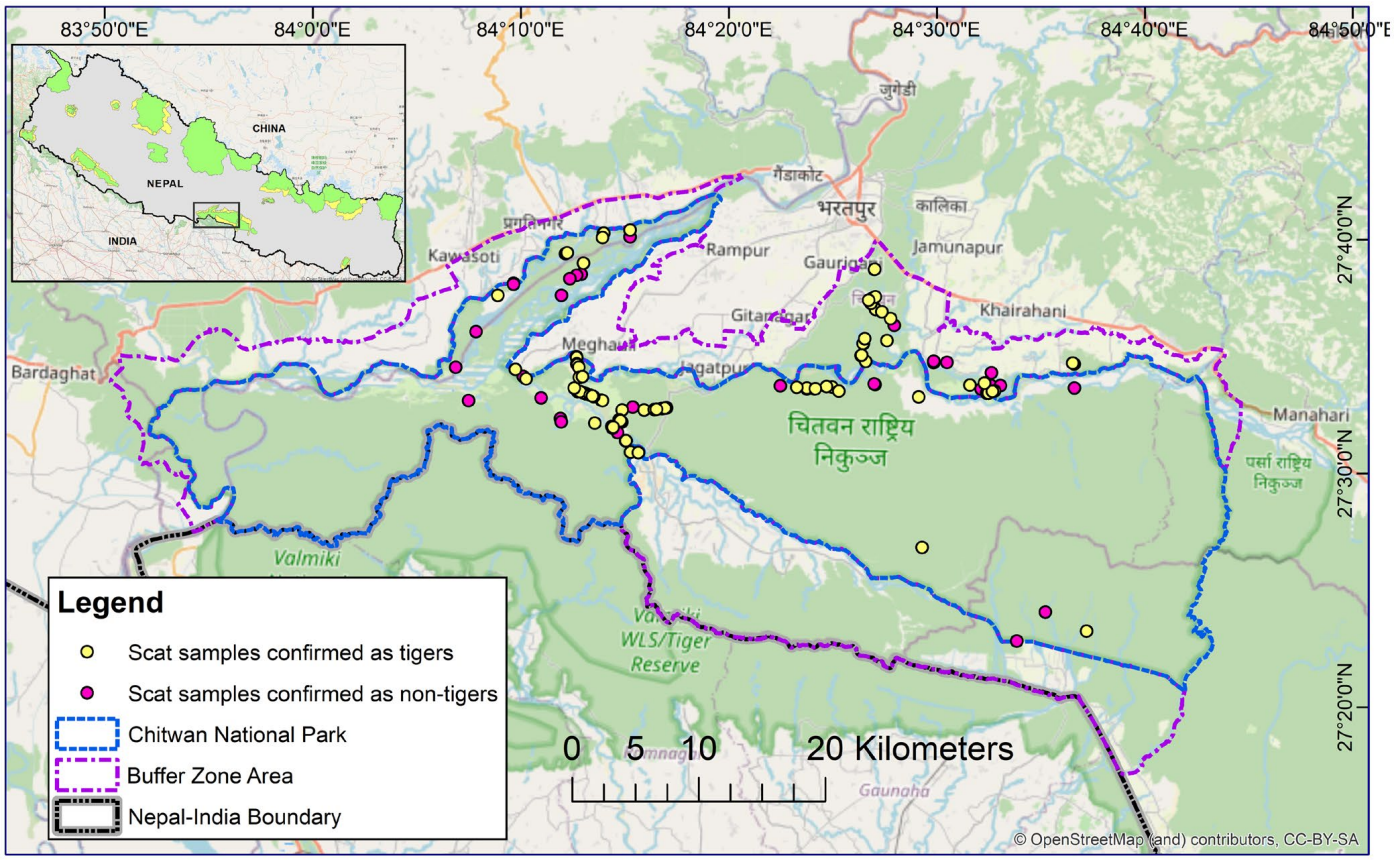
Study area of Chitwan National Park and its Buffer Zone Area, Nepal. The yellow dots show the samples confirmed as tiger scats (*n* = 81), and red dots show the samples confirmed as non‐tiger scats (*n* = 54) out of total scat samples (*n* = 135), collected from the national park and buffer zone during the dry season of 2024, overlaid on the study area.

Chitwan National Park, the first national park of Nepal, established in 1973 with an area of 952.63 km^2^, is situated in the lowland Terai of the southern part of central Nepal, having Parsa National Park of Nepal in the east and Valmiki Tiger Reserve of India in the south (Figure 1; DNPWC 2025). The park has a sub‐tropical climate with an average monthly maximum temperature between 24°C and 38°C, monthly minimum temperature between 11°C and 26°C; annual rainfall is about 2250 mm and 80% of the land is covered by Sal (*Shorea robusta*) Forest, Riverine Forest and Mixed Hardwood Forest, where 12% is grasslands and 3% is water bodies (Thapa 2011). The park holds the ox‐bow lakes and three major rivers– Narayani, Rapti, and Riu as the water sources for the wildlife. The park consists of diverse ecosystems ranging from the early succession stage of the alluvial floodplains to the climax forests on the foothills and slopes of the Churia physiographic range (Bhattarai and Kindlmann 2018). This park has the largest tiger population in Nepal, with approximately 36% of the country's total estimated 355 adult tigers (*n* = 128) (DNPWC and DFSC 2022). The prey species abundance in this park includes spotted deer, sambar deer, hog deer, barking deer, wild boar, and gaur (Karki et al. 2015).

The Buffer Zone (BZ) area around the national park was declared in 1996 comprising Barandabhar corridor forest, community‐managed forests and private agricultural land with an area of 729.37 km^2^ (DNPWC 2025). People in the area depend on agriculture and livestock, and rely on forests for fuelwood and fodder to support livelihoods. The buffer zone area is administered by the park authority in collaboration with local communities. The buffer zone has 22 buffer zone users' committees (BZUC) formed by the park to implement community development, biodiversity conservation, awareness, and forest resource management programs through local participation (Lamichhane et al. 2019). The buffer zone forests are legally accessible to buffer zone communities for resource use, which is also an extended habitat for wildlife, including tigers.

### 2.2 Field Collection and Predator Species Identification

We collected the scat samples opportunistically during the dry season (January–March) in 2024 from Chitwan National Park and its Buffer Zone forests, walking along the animal trails, forest roads, and riverbeds where tigers prefer to use and are likely to deposit scats (Smith et al. 1998) (Figure 1). The scats that resemble the morphological characteristics (size, appearance, lower degree of coiling) are preliminarily assigned as tiger scats, which were further identified by genetic analysis. Scat samples were collected in 50 mL Falcon Tubes and stored in a −20°C freezer in the laboratory of the Biodiversity Conservation Centre, Sauraha, Chitwan.

### 2.3 DNA Extraction and Species Identification

For DNA extraction, 200 mg of scat was scraped with a disposable blade and stored in a 1.5 mL Eppendorf tube at −20°C for further analysis. The DNA extraction from the processed scat samples was carried out using the Qiagen QIAamp DNA Mini Stool Kit (Qiagen, Germany) following the manufacturer's guidelines. Extraction blanks were included as a negative extraction to test for contamination.

As confirmation of carnivore is crucial where genetic analysis could be a significant approach to animal identification (Havmøller et al. 2021), we verified tigers' scat using tiger‐specific primers (TIF/TIR) that exclusively amplify tiger DNA at the 162 bp region of the mitochondrial *cytochrome‐b* gene on agarose gel electrophoresis (Bhagavatula and Singh 2006). The PCR was performed at a 20 μL total volume each using 1.30 μL each of 10 pmol/μl forward and reverse primers with 10 μL of 2× Qiagen master mix, nuclease‐free water 5.4 μL and 2 μL of template DNA. The thermocycling was performed at 95°C for 15 min followed by 35 cycles of 94°C for 30s; 59°C for 90s, and 72°C for 90s, with final extension at 72°C for 10 min. The amplified PCR products were visualized using 1.5% agarose gel electrophoresis, with the expected product size being 162 bp and were confirmed using a 100 bp ladder (Solis Biodyne).

### 2.4 Prey Profiling by DNA Metabarcoding Analysis

#### 2.4.1 Library Preparation for Illumina MiSeq Amplicon Sequencing

Amplification of prey species DNA in tiger‐positive samples was adapted with the general vertebrate primer 12SV5 (Riaz et al. 2011), which targets ~100 bp region of the vertebrate 12S rRNA gene. A blocking oligo primer was included to inhibit amplification of predator (tiger and leopard) DNA, which also enhances amplification of prey DNA. PCR was performed in a 25 μL total volume using 2.5 μL each of 1 pmol/μl forward and reverse primers with 12.5 μL of 2× Kapa HiFi Hot start ready mix, 5 μL of 10 pmol/μl PrioB blocking primer, and 2.5 μL of template DNA. The thermocycling was performed at 98°C for 3 min followed by 40 cycles of 94°C for 30 s, 60°C for 55 s. The amplified PCR products were visualized using 1.5% agarose gel at 90 V for 90 min. The PCR product was purified using a 0.6X ampure bead, and the library was quantified and normalized into equimolar concentration using Qubit 1X dsDNA High Sensitivity (HS). Libraries were prepared for scat samples, which were successfully amplified and gave a positive result with the tiger‐specific PCR, using the Nextera library preparation kit. Libraries were normalized to 4 nM along with 5% PhiX control and sequenced using MiSeq Reagent kit V2 (300 cycles) on an Illumina MiSeq platform (Illumina Inc., USA).

#### 2.4.2 Sequence Data Analysis and Prey Taxonomic Identification

Raw read quality was assessed using FastQC v0.11.9 (Andrews et al. 2010), and adapter sequences and low‐quality reads were filtered out with Trimmomatic v0.39 (Bolger et al. 2014). The trimmed reads were processed through the QIIME2 v2021.11.0 pipeline (Bolyen et al. 2019), where de‐noising was performed by further trimming, merging, and removing chimeric sequences with the DADA2 plugin. The processed reads were de‐replicated to generate unique sequence reads, or Amplicon Sequence Variants (ASVs), which were further filtered to contain only vertebrate taxa using BLAST similarity searches against the Vertebrate 12S reference database from NCBI GenBank. These target ASVs underwent taxonomic classification against the same reference database using the BLAST tool, with parameters set for query coverage at 0.85, percent identity at 0.97, maximum acceptance at 30, and minimum consensus at 0.51. The ASVs were clustered into taxonomic OTUs, and a taxonomic table showing sequence counts for each classified OTU across samples was created. Relative abundance for all samples was calculated on the basis of taxa, and a presence/absence table was generated, using a criterion that assigned prey as present if the relative abundance was greater than 1%, thereby filtering out rare reads resulting from sequencing artifacts.

### 2.5 Microscopic Hair Analysis (MHA)

After separating a portion of the sample for DNA analysis, the scats were washed through running tap water in a sieve of mesh size 0.5 mm and 0.1 mm in order to separate the prey remains (hairs, claws, and bones) and dried in sunlight for two to 3 days before microscopic examinations (Karanth and Sunquist 1995; Mukherjee et al. 1994). Scat samples were analyzed following the method used by (Ramakrishnan et al. 1999). From each reference sample, 40 hairs were randomly selected and analyzed using the reference guide by Bahuguna (2010) and the hair reference collections of wild prey and domestic prey species available in the laboratory.

To prepare the cuticular sample slides, 40 hairs from each sample were randomly selected, carefully dipped in a solution consisting of a 1:1 ratio of ethanol and diethyl ether in a petri dish for 15 min and then allowed to dry using tissue paper. Then a thin layer of transparent nail polish was applied to a glass slide, and the hairs (*n* = 20 of 40) were mounted on the polished surface. After the nail polish had dried (about 10 min), the hairs were carefully removed, leaving the imprint of cuticular scale patterns.

Similarly, for the preparation of medullary sample slides, hair plucked from the cuticular slides was used. These hairs were carefully dipped in the xylene solution for 30 min, dried, and placed on the slides.

The sample slides were then placed under the Olympus microscope at x400 magnification, where both the medullary structure and cuticular patterns were matched with the available reference slides. Additionally, pictures were taken with a Coslab Digital Camera (model: MDCE‐ 5C) for each hair imprint. Finally, for all scat samples of the tigers, the cuticular pattern of hairs was used to identify the prey species, whereas medullary sample slides were used to further validate the results.

### 2.6 Statistical Analysis

#### 2.6.1 Species Accumulation Curve

We assessed dietary richness and the number of scat samples needed to detect all prey species consumed by tigers in our study for both the DNA metabarcoding and the microscopic hair analysis (MHA) using prey accumulation curves. The statistical power of DNA metabarcoding (*n* = 58) and microscopic hair analysis (*n* = 78) was evaluated by comparing the total number of prey species present in the scat samples with 10,000 permutations (Thuo et al. 2019).

#### 2.6.2 Prey Species Occurrences, Preferences and Biomass Consumed

We summarized the prey species present in tiger scats and calculated the frequency of occurrence (FO%), percentage of occurrence (PO%), and the relative biomass consumed on the basis of both DNA metabarcoding and microscopic hair analysis. The FO% is calculated as the percentage of total samples in which the prey species were detected, whereas the PO% is the percentage of the number of occurrences of each prey species in relation to the total number of occurrences of all prey species.

We calculated the relative biomass consumed by tigers, adopted from Chakrabarti et al. (2016). With an asymptotic relationship for biomass consumed per collectable scat on prey weight, we used a generalized biomass model and found that the biomass consumed per collectable scat/predator weight was 0.033–0.025exp ^−4.284(preyweight/predatorweight)^ in our study.

We used Jacob's index (formula below) to calculate the prey preference of tigers (Jacobs 1974). The index ranges between +1 and −1, where +1 indicates maximum preferred and −1 least preferred.

$${\text{Jacob}}^{'}s\;\text{index}=\left(r-p\right)/\left(r+p-2\mathrm{rp}\right)$$

where *r* = proportion of biomass contribution by a prey species in the tiger scats, and *p* = proportion of prey biomass available for that species of the total prey population. The biomass available for tigers was calculated from prey density data obtained during the national tiger survey of Nepal, 2022. A line transect survey method was conducted to record the prey species in Chitwan National Park and adjoining forests, which were analyzed using the program DISTANCE version 7.2 to obtain density estimates of the prey species (spotted deer: 74.45 ind/km^2^ sambar deer: 7.9 ind/km^2^, hog deer: 2.15 ind/km^2^, wild boar: 5.03 ind/km^2^, barking deer: 2.94 ind/km^2^) (DNPWC and DFSC 2022).

To compare tiger diets between the national park and buffer zone, we used Fisher's Exact Test on the basis of the relative biomass of the prey species consumed. We carried out Fisher's Exact Test to compare the results of diet analysis on the basis of MHA and DNA metabarcoding.

## 3 Results

**FIGURE 2:**
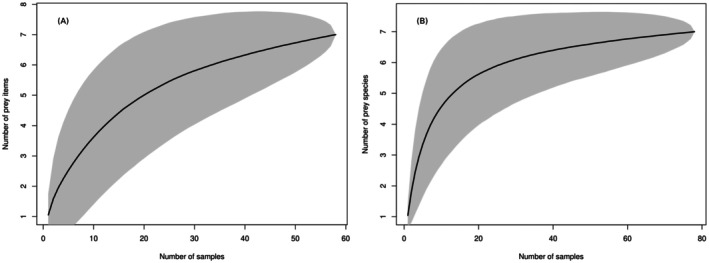
Prey species accumulation curve on the basis of DNA metabarcoding (A) and microscopic hair analysis (B) methods, showing the number of samples on the *x*‐axis and the number of identified prey species on the y‐axis. The species accumulation curve of microscopic hair analysis asymptotes at about 40 scat samples, whereas it does not reach the asymptote of DNA metabarcoding in 58 samples included in the analysis.

Of 135 scat samples collected (93 from the national park and 42 from the buffer zone), we were able to verify the defecator species of 98 scats with a result of these confirmed scat samples, sourced from different carnivore species resulting in 81 tigers, 13 leopards ( *Panthera pardus* ), two Felis spp., and one dog ( *Canis lupus familiaris* ). Out of 81 tiger scats confirmed, 23 samples contained only tiger DNA with no prey DNA, which was not used for diet analysis. Thus, 58 remaining tiger scat samples (38 from the national park and 20 from the buffer zone) were used for a diet analysis study in the DNA metabarcoding method. Although in the microscopic hair analysis method, of the 81 samples verified as tiger scat, three samples were discarded as they showed no clear signs of prey hairs or bones, so we used 78 tiger scat samples (50 from the national park and 28 from the buffer zone) to analyze the tiger diet. The species accumulation curve suggested that our samples collected represented the prey species available in the study area (Figure 2A,B).

### 3.1 Tiger Diet Composition and Prey Preference

A total of seven prey categories (species or taxa level information) were identified with varied species types and proportions in the tiger diet. DNA metabarcoding analysis revealed that spotted deer, sambar deer, hog deer, wild boar, monkey (family level), red jungle fowl, and rodent (*mus*) were found in tiger scats. The frequency of occurrence (FO%) and percentage of occurrence (PO%) of the prey species in the tiger diet via metabarcoding represented the highest with spotted deer (FO: 70.69%, PO: 67.21%, Biomass: 72.17%) followed by sambar deer (FO: 10.34%, PO: 9.84%, Biomass: 13.57%) and hog deer (FO: 10.34%, PO: 9.84%, Biomass: 8.78%) in Chitwan National Park and its buffer zone forests (Tables 1 and 2).

**TABLE 1:** Number of occurrences, frequency of occurrence (FO), and percentage of occurrence (PO) of prey species on the basis of DNA metabarcoding analysis scat samples (*n* = 58) of tigers in Chitwan National Park and its Buffer Zone Area, Nepal.

Prey items	Number of occurrences (N)			Frequency of occurrence (FO%)			Percentage of occurrence (PO)		
	National Park	Buffer zone	Total	National Park	Buffer zone	Total	National Park	Buffer zone	Total
Spotted deer ( *Axis axis* )	25	16	41	65.79	80	70.69	60.98	80	67.21
Sambar deer ( *Rusa unicolor* )	4	2	6	10.53	10	10.34	9.76	10	9.84
Hog deer ( *Axis porcinus* )	4	2	6	10.53	10	10.34	9.76	10	9.84
Wild boar ( *Sus scrofa* )	3	0	3	7.89	0	5.17	7.32	0	4.92
Monkey^a^ ( *Semnopithecus hector* )	1	0	1	2.63	0	1.72	2.44	0	1.64
Rodents^a^ (*Mus*)	3	0	3	7.89	0	5.17	7.32	0	4.92
Jungle fowl ( *Gallus gallus* )	1	0	1	2.63	0	1.72	2.44	0	1.64

^a^ Genus level information was obtained from DNA metabarcoding. In case of monkey, we assumed it as langur ( *Semnopithecus hector* ) after confirming from microscopic hair analysis.

**TABLE 2:** Relative biomass of prey consumed by tigers in Chitwan National Park and Buffer Zone Area on the basis of DNA metabarcoding of scat samples (*n* = 58) of tigers.

Prey species	Number of occurrences (N)	Mean body mass of prey (X) kg	Mean body mass of predator (Z) kg.	X/Z	Biomass consumed (Y) kg.	Corrected body mass of the predator (Y_c_)	A (%)	Relative biomass (D%)
Spotted deer ( *Axis axis* )	41	53	188	0.28	0.03	4.80	70.69	72.17
Sambar deer ( *Rusa unicolor* )	6	212	188	1.13	0.03	6.17	10.34	13.57
Hog deer ( *Axis porcinus* )	6	33	188	0.18	0.02	3.99	10.34	8.78
Wild boar ( *Sus scrofa* )	3	38	188	0.20	0.02	4.23	5.17	4.65
Langur ( *Semnopithecus hector* )	1	8	188	0.04	0.01	2.29	1.72	0.84
Rodents (*Mus*)	3	0.1	188	0.00	0.01	1.51	2.72	0.88
Jungle fowl ( *Gallus gallus* )	1	1	188	0.01	0.01	1.61	3.72	1.28

*Note:* X = Mean body mass of the prey (Karanth and Sunquist 1995; Bhattarai and Kindlmann 2012); Z = Mean body mass of the predator (Smith et al. 1983; Upadhyaya et al. 2018); Y = Biomass consumed; (Y = 0.033–0.025exp ^−4.284X/Z^), (Chakrabarti et al. 2016); Y_C_ = Y corrected for predator weight (Y × Z); A = The frequency of occurrence of the prey species in the scats; D = Relative Biomass consumed.

The microscopic hair analysis identified the same number of species but different species information. Specifically, spotted deer, sambar deer, hog deer, barking deer, wild boar, langur monkey, and rodents were observed in tiger scats collected. The frequency of occurrence (FO), percentage of occurrence (PO) and prey biomass were highest with spotted deer (FO: 37.18%, PO: 35.8%, and biomass: 37.03%) followed by sambar deer (FO: 25.64%, PO: 24.69%, and biomass: 32.82%) and hog deer (FO: 17.95%, PO: 17.28%, and biomass: 14.86%) (Tables 3 and 4). The results of the tiger diet between these two methodological approaches (DNA metabarcoding and microscopic hair analysis) were found to be significantly different (Fisher's Exact Test, *p*‐value = < 0.001). We used DNA metabarcoding results for further analysis of the tiger's prey preference and diet composition between the national park and buffer zone area.

**TABLE 3:** Number of occurrences, frequency of occurrence (FO), and percentage of occurrence (PO) of prey species calculated on the basis of microscopic hair analysis of tiger scat samples (*n* = 78) in Chitwan National Park and its Buffer Zone Area, Nepal.

Prey species	Number of occurrences (N)			Frequency of occurrence (FO%)			Percentage of occurrence (PO)		
	National Park	Buffer zone	Total	National Park	Buffer zone	Total	National Park	Buffer zone	Total
Spotted deer ( *Axis axis* )	15	14	29	30	50.00	37.18	28.30	50.00	35.80
Sambar deer ( *Rusa unicolor* )	13	7	20	26	25.00	25.64	24.53	25.00	24.69
Hog deer ( *Axis porcinus* )	10	4	14	20	14.29	17.95	18.87	14.29	17.28
Barking deer ( *Muntiacus muntjak* )	5	3	8	10	10.71	10.26	9.43	10.71	9.88
Wild boar ( *Sus scrofa* )	6	0	6	12	0.00	7.69	11.32	0.00	7.41
Langur ( *Semnopithecus hector* )	3	0	3	6	0.00	3.85	5.66	0.00	3.70
Rodents (*Mus*)	1	0	1	2	0.00	1.28	1.89	0.00	1.23

**TABLE 4:** Relative biomass of prey consumed by tigers in Chitwan National Park and Buffer Zone Area on the basis of microscopic hair analysis of scat samples (*n* = 78) of tigers.

Prey items	Number of occurrences (n)	Mean body mass of prey (X) kg	Mean body mass of the predator (Z) kg.	X/Z	Biomass consumed (Y) kg.	Corrected body mass of the predator (Yc)	A (%)	Relative biomass (D%)
Spotted deer *(Axis axis)*	29	53	188	0.28	0.03	4.80	37.18	37.03
Sambar deer *(Rusa unicolor)*	20	212	188	1.13	0.03	6.17	25.64	32.82
Hog deer *(Axis unicolor)*	14	33	188	0.18	0.02	3.99	17.95	14.86
Barking deer *(Muntiacus muntjak)*	8	19	188	0.10	0.02	3.16	10.26	6.72
Wild boar *(Sus scrofa)*	6	38	188	0.20	0.02	4.23	7.69	6.75
Langur *(Semnopithecus hector)*	3	8	188	0.04	0.01	2.29	3.85	1.83
Rodents *(Mus)*	1	0.1	188	0.00	0.01	1.51	1.28	0.40

*Note:* X = Mean body mass of the prey (Karanth and Sunquist 1995); (Bhattarai and Kindlmann 2012); Z = Mean body mass of the predator (Smith et al. 1983; Upadhyaya et al. 2018); Y = Biomass consumed; (Y = 0.033–0.025exp ^−4.284X/Z^), (Chakrabarti et al. 2016); Y_C_ = Y corrected for predator weight (Y × Z); A = The frequency of occurrence of the prey species in the scats; D = Relative Biomass consumed.

### 3.2 Prey Preference

**FIGURE 3:**
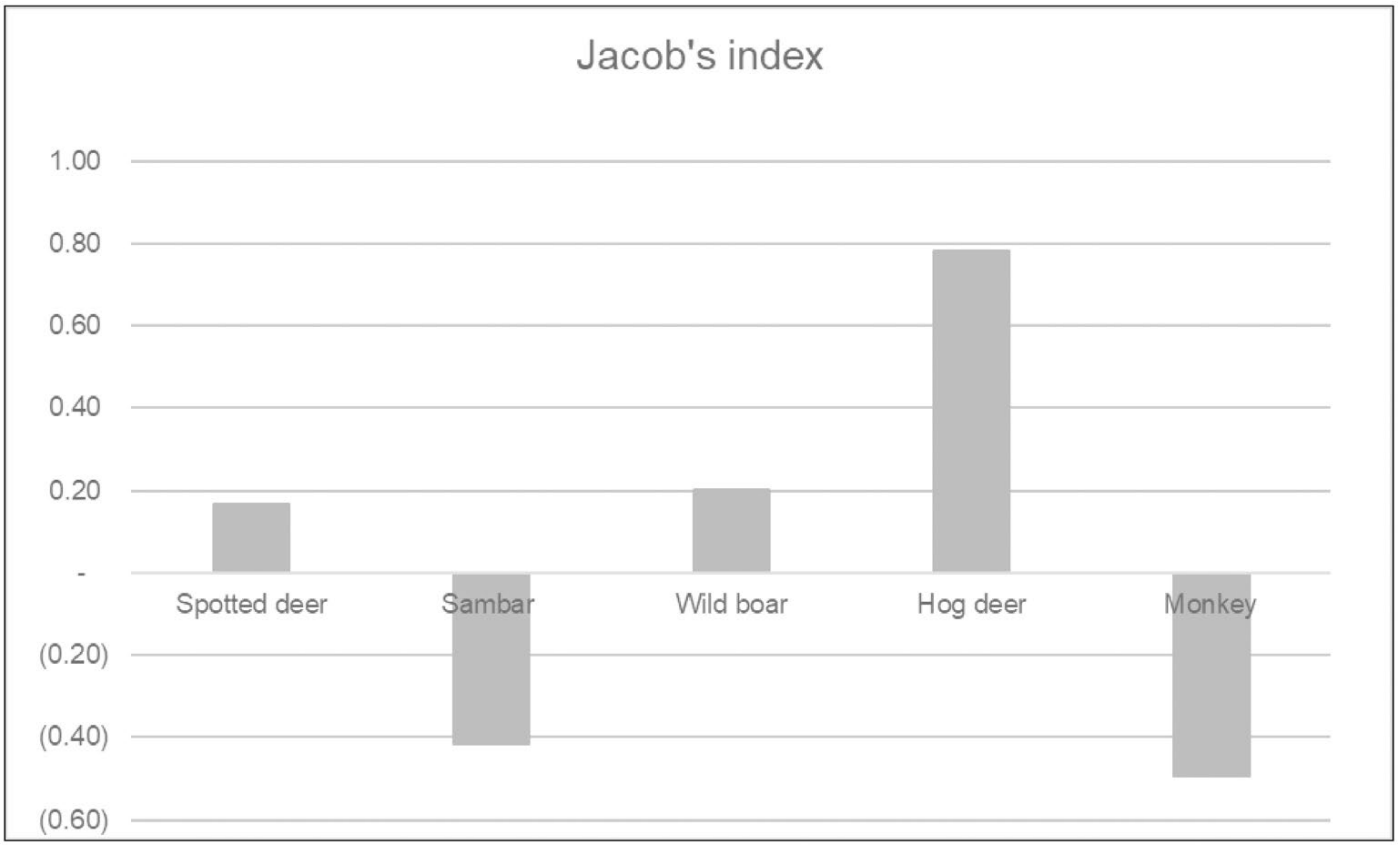
Jacob's index is calculated on the basis of the relative biomass consumed by tigers. We used DNA metabarcoding results of the tiger scats analysis. Jacob's index = (r−p)/(*r* + p−2rp), where *r* = proportion of biomass contribution by a prey species in the tiger scats and *p* = proportion of prey biomass available for that species of the total prey population. The index ranges between +1 and −1, where +1 indicates maximum preferred and −1 minimum preferred.

Jacob's index calculated on the basis of the relative biomass consumed by tigers showed that hog deer was the most preferred prey species, followed by wild boar and spotted deer proportionately upon their availability (Figure 3).

### 3.3 Differences in Tiger Diets Between National Park and Buffer Zone

The tiger diet via DNA metabarcoding discovered that the percentage of occurrence (PO) was highest with spotted deer (60.98%), followed by sambar deer (9.76%), hog deer (9.76%), wild boar (7.32%), langur (2.44%), mice (7.32%), and jungle fowl (2.44%) in the national park, whereas tiger diet percentage of occurrence (PO%) in the buffer zone area comprising spotted deer (80%), sambar deer (10%), and hog deer (10%), with exclusively dominated deer (Table 1). Tiger diet composition differed significantly between the national park and buffer zone (Fisher's Exact Test; *p*‐value = 0.023).

## 4 Discussion

We studied the diet composition of tigers in and around Chitwan National Park to dig out whether the domestic livestock contributed to their food. Microscopic hair analysis and molecular techniques were used to find out the accurate results. However, the frequency of occurrence (FO) and percentage of occurrence (PO) of prey items by two methods were slightly different; the relative biomass consumed by tigers was contributed by the prey species solely dominated by wild prey such as spotted deer, hog deer, sambar deer, and so forth, both in the core area and buffer zone of the park.

### 4.1 Diet Composition and Preference

We analyzed the tiger diet and prey preference in Chitwan National Park and Buffer Zone, Nepal, using both microscopic hair analysis and DNA metabarcoding methods for the first time. Seven prey taxa were detected in the tiger scats, comprising spotted deer, sambar deer, hog deer, wild boar, jungle fowl, rodents, and monkeys. Spotted deer had the maximum contribution (72.17%) to the relative biomass consumed by tigers, followed by sambar deer, hog deer, and wild boar. Spotted deer have the highest density in the park, thus readily available to tigers compared to other prey, which occur at relatively lower density (DNPWC and DFSC 2022). This finding is aligned with previous studies that identified the majority of the diet comprised of spotted deer in Chitwan National Park (Kapfer et al. 2011; Bhandari et al. 2017; Reynaert 2018). Similarly, the tiger diet studies in other protected areas (Parsa and Bardia national parks) in similar habitats also documented spotted deer contributing the highest proportion of the tiger diet (Upadhyaya et al. 2018; Lamichhane et al. 2022). In Sundarbans, Bangladesh, also, the spotted deer contributed a biomass of 78% to the tiger diet (Aziz et al. 2020).

However, the tiger's highest preference was found for middle‐sized prey, like hog deer, wild boar, and spotted deer. It is worth noting that tigers less prefer large‐sized prey like sambar deer despite their high energetic return (killing a sambar). Furthermore, tigers likely prey on sambar deer less often, potentially because of their dispersed population and low density, requiring more energy to hunt. This result is also consistent with other previous studies in which tigers prefer to hunt medium‐sized prey in Bardia, Nepal; (Støen and Wegge 1996; Odden and Wegge 2007; Wegge et al. 2009) and TAL in India (Ranjan et al. 2024). Additionally, being solitary in nature (Odden and Wegge 2007), hog deer are easy to hunt compared with colonial deer such as spotted deer.

### 4.2 Diet Comparison in National Park and Buffer Zone Area

The tiger diet composition is significantly different between the national park (with prey taxa = 7; spotted deer, sambar deer, hog deer, wild boar, jungle fowl, rodents, and monkeys) and the buffer zone area (with prey taxa = 3; spotted deer, hog deer, and sambar deer). Spotted deer represented the highest percentage of occurrences in both areas, whereas wild boar, rodent, monkey, and jungle fowl were detected in the tiger diet only in the national park area.

Notably, we did not find any sign of livestock in the scat samples collected in both the national park and buffer zone forests, suggesting tigers prefer consuming wild prey for their diet in a natural setting. However, the human‐tiger conflict, particularly livestock predation, is frequently found in the buffer zone of the national park (Dhungana et al. 2018). These findings were also aligned with a previous study in and around Chitwan National Park by Reynaert (2018), who also identified that the major contribution to the tiger diet was from wild prey, even in the buffer zone forest and corridor forests.

A study on conflict‐causing tigers (Lamichhane et al. 2017) shows that only a few individuals (< 5% of the tiger proportion) are involved in conflict, which is very few in comparison to the total tiger population, and thus, we may have missed the scat of such individuals when they consumed livestock. The park authority rescued six tigers from the buffer zone area, which came into conflict with local people (CNP 2024). Previous studies in Chitwan suggested that old and injured tigers relied more on easy prey, including livestock on the forest edge (Lamichhane et al. 2017). However, problem tigers were proactively captured. Generally, tigers prefer habitats with abundant prey and less human disturbance (Bhattarai and Kindlmann 2018). Having tigers with abundant natural prey in the buffer zone forest and corridors may not result in livestock predation if we proactively manage the injured and old tigers.

### 4.3 Comparison of DNA Metabarcoding and Microscopic Hair Analysis Methods

Both DNA metabarcoding and microscopic hair analysis identified an equal number of prey taxa (*n* = 7); six species were common. However, the contribution of prey species to the diet of tigers was mostly dominated by spotted deer, followed by sambar deer and hog deer in both methods; frequency of occurrences and relative biomass of these species varied between techniques. This is mostly due to differences in technological advancement between the methods, as microscopic hair analysis relies more on observation, whereas DNA metabarcoding is an advanced and primer‐based identification approach (Havmøller et al. 2021). This advancement in technique helped to confirm the presence of certain species. For example, jungle fowl was identified only in DNA metabarcoding, and barking deer species were identified in microscopic hair analysis, not in DNA metabarcoding. Such differences in diet composition analysis between the two methods indicated better performance of DNA metabarcoding in species identification. We suggest that species confirmation for those species having similar morphological hair structure should be cross‐validated with DNA metabarcoding for actual identification. The prey species biomass consumption estimate varied between MHA and DNA metabarcoding. A study on the hair morphology of ungulate prey species suggested that barking deer hair has a similar pattern to spotted deer but contrasts with sambar deer (Desai et al. 2019). This increases the chances of misidentification of species while observing the structure of hair in microscopes. Thus, we relied on DNA metabarcoding results for further analysis in this study.

Additionally, we applied only one widely used marker (12S marker) because of resource constraints in this study. The 12S marker has been widely used in vertebrate‐focused metabarcoding studies because of its short amplicon length (~100 bp) and is well‐suited for degraded DNA from non‐invasive samples (Riaz et al. 2011). However, the short length also limits taxonomic resolution, particularly among closely related species such as those within the Cervidae family in our study system. Despite this limitation, the 12S marker allowed us to distinguish among key prey species, including spotted deer, hog deer, and sambar deer, which are also known from previous studies using microscopic prey identification methods as among the most frequently consumed prey of the tiger. However, employing multiple primer sets targeting different markers could improve taxonomic resolution, as well as methodological improvement in metabarcoding in cases where certain species share identical sequences at one locus but can be differentiated at another (Havmøller et al. 2021).

## 5 Conclusions

The results of this study provide an in‐depth understanding of the diet of tigers in the national park and the buffer zone area, which explicitly relied on wild prey, while the tiger population was recovering in Nepal. The tiger was maximizing its hunting efficiency by targeting solitary, medium‐sized prey such as hog deer and wild boar for their easy availability and muscular biomass. Thus, park authorities should focus on the conservation of not only large prey but also medium‐sized prey for long‐term tiger conservation to accommodate more tigers up to their ecological carrying capacity and reduce the risk of livestock predation. However, the findings extracted from small scat samples of the confirmed tiger scats for DNA metabarcoding (*n* = 58), particularly in the buffer zone (*n* = 20), collected in only one season (dry period) of the year might not represent the broader prey assemblage of the area (both natural and domestic). Literature suggests that the minimum value needed for comparative analysis would be ~90 scat samples (Trites and Joy 2005). As only a few individual tigers (< 5% of the population) are involved in conflict, and the park authority proactively captures the conflict‐causing tigers, we might miss the scats of those tigers. Furthermore, the limited samples might result in a low frequency of occurrences of larger prey like sambar, and this may also be due to its lower density than spotted deer in the area. We recommend further study with more sample sizes, capturing both seasons (dry and wet) of the year, which may yield a better understanding of the tiger's diet composition in the area. Even collecting scats from outside of the protected areas (from the corridor) may also provide insight into the diet of the dispersing tigers.

## Author Contributions

**Hari Bhadra Acharya:** conceptualization (equal), data curation (equal), formal analysis (equal), funding acquisition (equal), methodology (equal), project administration (equal), resources (equal), writing – original draft (equal). **Laura D. Bertola:** writing – original draft (equal), writing – review and editing (equal). **Dinesh Neupane:** data curation (equal), formal analysis (equal), writing – original draft (equal). **Babu Ram Lamichhane:** formal analysis (equal), writing – original draft (equal), writing – review and editing (equal). **Herwig Leirs:** supervision (equal), writing – original draft (equal), writing – review and editing (equal). **Prajwol Manandhar:** formal analysis (equal), writing – original draft (equal). **Hans H. de Iongh:** conceptualization (equal), supervision (equal), writing – original draft (equal), writing – review and editing (equal).

## Funding

Funding for this research was provided by WWF Nepal for field data collection of scat samples (Grant Agreement No. NPl 7775), and from the Leo Foundation, Rohgorst 343, 6708 KX Wageningen, Netherlands, for DNA metabarcoding.

## Disclosure

Statement of generative AI: The authors declare no use of generative AI and AI‐assisted tools.

## Ethics Statement

The study abides by all the required legal and ethical standards during scat collection and manuscript preparation.

## Conflicts of Interest

The authors declare no conflicts of interest.

## Data Availability

The data of the diet results from both microscopic hair analysis and DNA metabarcoding are available in Figshare: https://doi.org/10.6084/m9.figshare.29822087.
